# Subsequent Actions Engendered by the Absence of an Immediate Response to the Proposal in Mandarin Mundane Talk

**DOI:** 10.3389/fpsyg.2022.942266

**Published:** 2022-08-01

**Authors:** Quanxi Hao, Hui Guo, Chuntao Li, Shuai Yang

**Affiliations:** ^1^School of Foreign Languages, Shanxi University, Taiyuan, China; ^2^Department of Foreign Languages, Taiyuan University, Taiyuan, China

**Keywords:** proposal, silence, subsequent actions, deontic stance, deontic trends

## Abstract

When there is no immediate response after a proposal and normally the silence is longer than 0.2 s, the proposer would take subsequent actions to pursue a preferred response or mobilize at least an articulated one from the recipient. These actions modulate the prior deontic stance embedded in the original proposal into four trends as follows: (1) maintaining the prior deontic stance with a self-repair or by seeking confirmation; (2) making the prior deontic stance more tentative by making a revised other-attentiveness proposal, providing an account, pursuing with a tag question, or requesting with an intimate address term; (3) making the prior deontic stance more decisive by making a further arrangement (for the original proposal), closing the local sequence, or providing a candidate unwillingness account (for the recipient's potential rejection); and (4) canceling the prior deontic stance by doing a counter-like action. Additionally, these trends inherently embody a decisive-to-tentative gradient. This study would penetrate into the phenomena occurring in Mandarin mundane talk with the methodology of Conversation Analysis to uncover the underflow of deontic stance.

## Introduction

In talk-in-interaction, participants take turns to talk with minimal gap and overlap. Operations of the turn-taking follow such rules as Rule 1a, 1b, and 1c, and Rule 2^1^,^1^ (Sacks et al., 1974). However, it is accountable if Rule 1a fails, or the recipient fails to take the turn to give a coherent response (Pomerantz, 1984). For instance, the recipient may look blank or questioning, or make hesitating noises such as *Uhs, Ums*, and *Wells*. The recipient's failure would be treated as having some problem in responding or as projecting a high probability of a dispreferred response since a preferred one will normally be produced immediately (Pomerantz and Heritage, 2013). At this moment, the speaker would pursue a response by “clarifying, reviewing the assumed common knowledge, and modifying one's position” (Pomerantz, 1984: 153). This study focuses on similar phenomena in proposal sequences in Mandarin mundane talk. It is found that in most cases when the recipient fails to make “an immediate response” (Lee, 2013: 417) to a proposal and generally the duration of silence^2^ is longer than 0.2 s (Stivers et al., 2009; Roberts et al., 2015), the proposer would take subsequent actions to pursue a preferred response or mobilize at least an articulated one from the recipient.

As a social action, proposing is different from other social actions such as requesting, offering, inviting, or suggesting, and “proposing invokes both speaker and recipient in (a) the decision task and (b) the ensuing activity in a way that is mutually beneficial” (Stivers and Sidnell, 2016: 148). Prior studies on proposal sequences generally focus on (1) actions prior to the proposing turn (Drew, 1984; Couper-Kuhlen, 2014; Robinson and Kevoe-Feldman, 2016); (2) the initial actions of proposing (Drew, 2013; Stevanovic, 2013; Toerien et al., 2013; Couper-Kuhlen, 2014; Kushida and Yamakawa, 2015; Robinson and Kevoe-Feldman, 2016; Stevanovic and Monzoni, 2016; Stivers and Sidnell, 2016; Stevanovic et al., 2017; Stivers et al., 2017; Yu and Hao, 2020; Thompson et al., 2021); (3) responses to a proposal (Davidson, 1984; Heritage, 1984a; Stevanovic, 2012b; Stevanovic and Peräkylä, 2012; Ekberg and LeCouteur, 2015; Stevanovic and Monzoni, 2016); and (4) subsequent actions after a response to a proposal (Stevanovic, 2012a; Maynard, 2016). For example, Drew (1984: 146) concluded that if a speaker wishes to invite a recipient to come over or do something together, one of the options available is “to hint at an opportunity for some sociability, and leave it to the recipient to propose an arrangement explicitly.” Stivers and Sidnell (2016: 148) examined two common ways that speakers propose a new joint activity with “*Let's X*” and “*How about X*,” in which “*Let's* constructions treat the proposed activity as disjunctive with the prior, while *How about* constructions treat the proposed activity as modifying the ongoing activity.” Additionally, besides an affirmative response token, “a second unit of talk is required where the recipient indexes her stance toward the fulfillment of the remote proposal” (Lindström, 2017: 142). Although when encountering a potential or an actual rejection, a proposer “may then display an attempt to deal with this possibility or potentiality through the doing of some subsequent version” (Davidson, 1984: 124, 125).

Moreover, it is observed that the subsequent actions or versions conducted by the proposer in this study are highly related to the deontic stance, which refers to the display of “the capacity of an individual to determine action” (Stevanovic, 2018: 1). In addition, these actions modulate the prior deontic stance embedded in the original proposal into four trends as follows: (1) maintaining the prior deontic stance with a self-repair or by seeking confirmation; (2) making the prior deontic stance more tentative by making a revised other-attentiveness proposal, providing an account, pursuing with a tag question, or requesting with an intimate address term; (3) making the prior deontic stance more decisive by making a further arrangement (for the original proposal), closing the local sequence, or providing a candidate unwillingness account (for the recipient's potential rejection); and (4) canceling the prior deontic stance by doing a counter-like action. Additionally, these trends inherently embody a decisive-to-tentative gradient and are analyzed in the following sections through related sequences. By examining the actual production of proposers' subsequent actions, we hope to uncover the underflow of deontic stance modulated in and through proposal sequences.

## Materials and Methods

Using everyday telephone talks in Mandarin Chinese as research materials, this study adopts the method of Conversation Analysis (hereafter CA) to investigate the occurrence of subsequent actions in proposal sequences.

“The central domain of data with which conversation analysts are concerned is everyday, mundane conversations” (Heritage, 1984a: 238). The whole database from which targeted proposal sequences are selected consists of 662 intact Mandarin mundane telephone talks (33 h, 35 min, 44 s) collected during 2014–2022 among classmates, friends, lovers, couples, relatives, and parent-child, out of which 112 intact telephone calls (4 h, 59 min, 36 s) contain 226 proposal sequences. Then, 34 targeted proposal sequences have been selected, which include the phenomena under investigation. All the data are transcribed according to CA conventions (Hepburn and Bolden, 2013).

One important research focus of CA is social action (Drew, 2013), which is implemented on a turn-by-turn basis in conversation. Most basically, an action sequence consists of a first pair part (FPP) and a second pair part (SPP), and the action enacted by an FPP normatively requires one of the alternative types of responsive actions by an SPP (Schegloff, 2007). For example, the recipient may accept or reject a proposal, or fail to respond to it. This study examines the recipient's failure to respond to a proposal. In facing the recipient's absence of an immediate response to a proposal, the proposer would take subsequent actions to solicit or mobilize a preferred or vocal response from the recipient. These actions modulate the prior deontic stance.

Table 1 shows the modulated deontic trends and the distribution of these subsequent actions.

**Table 1:** Deontic trends and subsequent actions in proposal sequences.

**TRENDS**	**ACTIONS**	**NO**.
(1) Maintaining the prior deontic stance	Doing a self-repair	5
	Seeking confirmation	2
(2) Making the prior deontic stance more tentative	Making a revised other-attentiveness proposal	7
	Providing an account	6
	Pursuing with a tag question	7
	Requesting with an intimate address term	1
(3) Making the prior deontic stance more decisive	Making a further arrangement	3
	Closing the local sequence	1
	Providing a candidate unwillingness account	1
(4) Canceling the prior deontic stance	Doing a counter-like action	1

## Subsequent Actions Engendered by the Absence of an Immediate Response

The proposer would do a self-repair or seek confirmation to clarify the original proposal or reexamine the assumed common knowledge in the first trend, or make a revised other-attentiveness proposal to modify his/her position in the second trend, to pursue a preferred response or at least mobilize an articulated one in this research. These solutions to the absence of an immediate response are identical to the findings of Pomerantz (1984) on assertions. However, more subsequent actions have been identified in proposal sequences in this research. In addition, they will be illustrated with examples in the following sections.

### Maintaining the Prior Deontic Stance

There are usually two ways to maintain the prior deontic stance, which are doing a self-repair and seeking confirmation. They are commonly conducted by the proposer to fix the interactional problems in terms of the speaker's or the recipient's epistemic domain (Heritage, 2013). By doing so, the proposer not only deals with the potential interactional problems but also provides the recipient with another chance to respond in the face of a growing silence, which may indicate an impending rejecting and disaffiliating response (Sacks et al., 1974).

#### Doing a Self-Repair

When engaging in a conversation, interactants frequently encounter problems in hearing, speaking, and understanding. Under such circumstances, the conversational repair is resorted to by interactants to ensure “that the interaction does not freeze in its place when trouble arises, that intersubjectivity is maintained or restored, and that the turn and sequence and activity can progress to possible completion” (Schegloff, 2007: xiv). In addition, there is “a strong empirical skewing” (Schegloff et al., 1977: 362) toward self-repair than other-repair.

In example (1), Liang and Li are friends and college students. Liang has promised to lend her library card to Li, and now they are making an arrangement to transfer the card.

**Table:** 

(1)	**14LJ_JKJM**	
39	Liang: →	*yaobu- yaoburan ni gen women yikuai chifan ba me*. Otherwise- Otherwise you and us together eat PRT PRT. **Or you can have a meal with us together**.
40		(1.0)
41	Liang: →	*wo:. [e:, Liu he Shi*. Me:. [Uhm:, Liu and Shi. **Liu, Shi and me**.
42	Li:	*[e, hai you na shui le*. [Uhm, Also have that who PRT. **There is also one person**.
43	Liang:	*en*. Yeah. **Yeah**.
44	Li:	*.h hai you Laogong le. wo gen Laogong qu ya*. .h also have Laogong PRT. I follow Laogong go PRT. **There's also Laogong. I'll eat with Laogong**.

After they decide to meet each other at Liang's dormitory, from which Li is distant (data not shown), Liang proposes having a meal together and transferring the card passingly, thus reducing Li's cost. However, no verbal response is produced but occurs a noticeably long silence (1.0 s) in line 40 that may indicate a certain difficulty for Li. Then, in line 41, Liang self-repairs the pronoun “*women*” in line 39 with “*wo:. e:, Liu he Shi*.” (Schegloff et al., 1977), which indicates that Liang treats the occurrence of no immediate response as the result of her ambiguous expression in line 39 since the identity of “*women*” should be established “at the time the pronoun is used” (Li and Thompson, 1989: 132). Therefore, what Liang is doing with the self-repair is to inform Li of the specific ones having a meal together to address the possible interactional problems, instead of modulating the prior deontic stance embedded in the original proposal. In this regard, doing a self-repair does not impose Li to accept the proposal, and the prior deontic stance is maintained.

#### Seeking Confirmation

By seeking confirmation, the proposer displays his/her relatively low epistemic stance compared with the recipient (Heritage, 2013). In this way, the recipient has been involved to reexamine the assumed common knowledge related to the original proposal.

In the following example, the husband (Wei) and his wife (Jiu) are discussing how to accomplish the wife's eye-brow shaping and their lunch arrangement with their child.

**Table:** 

(2)	**18H_XMCF**	
28	Wei: →	*e:, wo shi zai xiang*, Uhm:, I am at thinking, **I am considering that**,
29	→	*Yaobu jiu shi zanmen dai shang haizi*, Otherwise just be we take up kid, **Or we could take our kid**,
30	→	*wo:, wo he haizi dengde ni*. I:, I and kid wait you. **The kid and I will wait for you**.
31	→	*women wande, (0.6) ni xiumei me*. We play, (0.6) you eye-brow shaping PRT. **We play while you do eye-brow shaping**.
32	→	*xiumei wan le zanmen zai:*, eye-brow shaping finish PRT we again:, **After finishing eye-brow shaping we then**,
33	→	*song guoqu haishi za ya*. drive back or what PRT? **drive our kid back or what else do you think?**
34		(1.6)
35	Wei: →	*ni shi yao xiumei le wa*. You do want eye-brow shaping PRT PRT. **You do want eye-brow shaping, don't you?**
36		(0.3)
37	Jiu:	*ao*. Yeah. **Yeah**.

In the talk before line 28 (data not shown), they have already talked about other arrangements but not fully agreed with each other. Then in lines 28–33, the husband makes another proposal, yet the wife does not respond immediately. Instead, a silence of 1.6 s in line 34 occurs. Through confirmation seeking in line 35 to check if his proposal's premise is valid or if the wife's eye-brow shaping is still on her “wish list,” the husband treats the silence as an interactional problem. Therefore, this action serves to make the husband see if the original proposal is appropriate, instead of modulating the prior deontic stance. In this regard, seeking confirmation does not impose the wife to accept the proposal, and the prior deontic stance is maintained.

In summary, by doing self-repair or seeking confirmation, the proposer creates another opportunity for the recipient to provide a preferred response or at least an articulated one, thereby solving the discontinuity in the ongoing talk and maintaining the prior deontic stance.

### Making the Prior Deontic Stance More Tentative

According to the data, the proposer produces four kinds of subsequent actions to make the prior deontic stance more tentative. These actions include making a revised other-attentiveness proposal, providing an account, pursuing with a tag question, and requesting with an intimate address term.

#### Making a Revised Other-Attentiveness Proposal

Speakers could conduct a self-repair with “I-mean utterance” (Maynard, 2016: 74) to display other-attentiveness. For example, in a proposal sequence, the speaker produces a repair-formatted utterance “to shift attention from the speaker and his desires, to the recipient and his or her needs or experiences” (Maynard, 2016: 88, 89). Similarly, in dealing with an absence of an immediate response in an assertion sequence, the speaker would change his/her position from the one (s)he had just asserted with the remedy that “apparently is directed toward a problem caused by the speaker having said something that was wrong” (Pomerantz, 1984: 162). In proposal sequences in this research, the proposer would revise his/her original proposal when there occurs a noticeably long silence. In addition, the above three findings all focus on the similar phenomenon of other-attentiveness occasioned by the silence or the delay which “is a general device which permits potentially ‘face-threatening' rejections to be forestalled by means of revised proposals, offers and the like” (Heritage, 1984a: 275, 276).

In example (3), Yao and Li are friends. They have both taken part in a Special Offer launched by a bank, which is supposed to return a certain amount of money to their phone bills in 15 days. However, they have not received any money after waiting for more than 15 days. Since Yao has called the head office of the bank, Li asks for more information about it. Then, Yao tells Li the solution given by a bank staff, which obviously cannot explain the delayed date or provide an exact date of returning the money (data not shown).

**Table:** 

(3)	**14DY_HFDY**	
176	Li:	*na- zanmen zhe dou duoshao tian la*. Then- we this already how many day PRT. **How many days have we already waited?**
177		*ta shuo shiwuhao yihou yiyiyiqian me*, He said 15^th^ after bebebefore PRT, **He said before 15**^**th**^,
178		*xianzai dou ershiyihao la*. now already 21^st^ PRT. **now it's already 21**^**st**^.
179	Yao:	*ao::*. Oh::. **Oh**.
180		(0.8)
181	Yao:	*nao bu qing*. Figure not clear. **Can't figure it out**.
182		(0.3)
183	Yao: →	*jiu dengde wa*. Just wait PRT. **Then just wait**.
184		(0.9)
185	Yao: →	*huozhe shi natian*, Or be someday, **Or someday**,
186	Li:	*ao*. Oh. **Oh**.
187		(0.2)
188	Yao: →	*zan wenwen:: jiu zhege zhege Fenhang*. we ask ask:: just this this Bank Branch. **Let's ask the Bank Branch**.
189		(0.9)
190	Li:	*°ao:.° ao. na zhi neng- Ұhuoji xianzai hai qiande fei le.Ұ* °Ok:.° Ok. Then only could- ¥Brother now still overdue fee PRT.¥ **Ok. That is the only way. My phone bill now is still overdue**.
191		*Ұjiu dengde ta gei wo wanghuifan le.Ұ* ¥Just wait it give me back PRT.¥ **Just wait it to send the money back to me**.

In lines 176–178, Li complains about the delaying date and related issues since “part of how a complaint is formed is to provide for the recognizability of the offender's wrongdoings” (Pomerantz, 1986: 221). In responding to Li's complaint, Yao acknowledges it merely with a minimal response in line 179 without doing any other action. In addition, there is a silence of 0.8 s in line 180, which probably projects disaffiliation from Li. Then, Yao continues to provide an account or a disclaimer in line 181 informing that he cannot figure it out. Yet after another silence of 0.3 s in line 182, Yao proposes waiting to solve the problem in line 183, which follows a silence of 0.9 s in line 184 indicating that Li is not satisfied with the proposal. After the silence, Yao revises his proposal to call the branch some day in lines 185 and 188. In addition, Li gives his response in line 190, which indicates that the revised proposal in line 188 is more acceptable. Therefore, when the recipient fails to provide an immediate response, the proposer would revise the original proposal to make it more other-attentive. In doing so, the recipient is provided with a new opportunity to respond and also faces less pressure or imposition to accept the revised proposal. Thus, the prior deontic stance is modulated to be more tentative.

#### Providing an Account

An account can be provided by speakers to “modify (e.g., change, explain, justify, clarify, interpret, rationalize, (re)characterize, etc.), either prospectively or retrospectively, other interlocutors' understandings or assessments of conduct-in-interaction in terms of its ‘possible' breach of relevance rules” (Robinson, 2016: 15, 16). Therefore, when there is a dispreferred response or projection of a dispreferred response, the speaker would provide an account to make his/her proposal more justified and easier to be accepted. Thus, the prior deontic stance is modulated to be more tentative, and the imposition embedded in the original proposal is downgraded.

In example (4), Wang and Han are classmates and friends. Wang is calling Han to talk about the driving license test. After Han confirms that the test will be arranged only if they are sure to have a score of 95 (a full score is 100) in the practice test, Wang expresses her worry about it (data not shown).

**Table:** 

(4)	**15HB_MNKS**	
218	Han:	*ni- ni- ni kan le mei?* You- You- You review PFV not? **Have you already reviewed for the test?**
219	→	*yaobu, .h zanmen xiawu xian qu kanyikan*, *wanle nage sha*. Otherwise, .h We afternoon first go have a look, after that one what. **Or let's go there and have a look this afternoon, then it depends**.
220		(0.5)
221	Han: →	*zuiqima zhidao- (0.4) zuiqiama renjia zhidao ni qu le shi ba*. At least know- (0.4) At least they know you go PRT be PRT. **At least they know you've been there, right**.
222		(0.6)
223	Wang:	*ao. Xing. Xing*. Yeah. Ok. Ok. **Yeah. Ok. Ok**.

After asking if Wang has reviewed what is related to the test in line 218, Han proposes in line 219 that they can have a look at the driving license school. In addition, immediately after, there occurs a silence of 0.5 s in line 220. What Han is doing after the silence is providing an account in line 221, which makes her proposal more convincible and more explicitly other-attentiveness, since Han mentions that they would better let the officers know their presence at the driving license school, which would probably indicate a positive attitude toward the test from the perspective of the officers. In doing so, the proposer attempts to make the original proposal easier to be accepted, which makes the prior deontic stance more tentative rather than more decisive.

#### Pursuing With a Tag Question

“A statement can become a question by the addition of a short A-not-A question form of certain verbs as a tag to that statement” (Li and Thompson, 1989: 546). Tag questions can not only transform a statement into an interrogative in a proposal sequence but also decrease the deontic stance embedded in the original proposal in the meantime.

In example (5), Xing and Ying are friends, and Xing is calling Ying to meet her at the hotel as they have planned since Ying has booked the hotel rooms for Xing and another friend. However, Ying is still at home right now when Xing and the other friend are already on the way to the hotel in a taxi. Then comes the following talk.

**Table:** 

(5)	14JY_JMJH	
21	Ying:	*ni dade che:?* You take taxi:? **You are taking a taxi?**
22		(0.6)
23	Xing:	*en.=* Yeah.= **Yeah**.
24	Ying: →	*=.h ai. Xing- Xingxing na ni dade che*, =.h Alas. Xing- Xingxing then you take taxi, **Alas. Xingxing, since you are taking a taxi**,
25	→	*ni zhede wa, ni gao ta dao Julun*, you this way PRT, you tell him go Julun, **you can do it like this, you tell him to go to Julun**,
26	→	*.h Paichusuo menkou, ranhou wo gei ni song chuqu*, .h Police Station gate, then I give you deliver out, **at the gate of the Police Station, then I'll deliver it out to you**.
27		(0.5)
28	Ying: →	*xing bu xing?* Ok not ok? **Ok?**
29	Xing:	*<Julun Paichusuo?>* <Julun Police Station?> **Julun Police Station?**
30	Ying:	*en. Beidajie Julun Paichusuo*. Yeah. Beidajie Julun Police Station. **Yeah. Beidajie Julun Police Station**.
31		(0.8)
32	Xing:	*ao*. Oh. **Oh**.

After making sure that Xing is in a taxi right now through lines 21 and 23, Ying proposes handing over the hotel receipts in a place, which is convenient to them in lines 24–26. However, there occurs a silence of 0.5 s in line 27, after which Ying pursues Xing's response with a tag question “*xing bu xing?*” in line 28. This tag question is meant to mobilize the addressee to make a preferred response or at least an articulated one. After the production of the other-initiated self-repair sequence (partial repeat) (Robinson and Kevoe-Feldman, 2010) in lines 29–30 and the silence (0.8 s) in line 31, Xing responds to Ying with merely an acknowledgment token (Heritage, 1984b) in line 32. The tag question in line 28 makes a potential inter-turn gap into an intra-turn pause (Schegloff, 2016). The inter-turn gap indicates that the recipient fails to produce an immediate response where (s)he is interactionally expected to give one. Thus, by pursuing with a tag question, the proposer makes the silence something that is caused by him/herself, and the recipient acquires a new chance to respond. Besides, the tag question in line 28 works to display more contingency (Curl and Drew, 2008) to the recipient's acceptance compared with the original proposal without a tag question, thus making the prior deontic stance more tentative.

Besides the employment of “*xing bu xing* (similar to ‘ok')” in line 28 in example (5), there are other tag questions or similar tags occurring in the data, which include “*zen me yang* (what do you think),” “*ha* (ok),” “*ke yi wa* (is that ok),” “*shi bu shi* (similar to ‘right'),” “*a* (right),” and “*hao ba* (ok)”. In addition, they all function to make the prior deontic stance more tentative and more or less downgrade the imposition displayed in the original proposal.

#### Requesting With an Intimate Address Term

As put forward by Sacks (1964) in his lecture, the Membership Inference-Rich Representative Device (M.I.R.) means that “a great deal of the knowledge that members of a society have about the society is stored in terms of these categories” (Jefferson, 1989: 90). Also, “When people recognize someone as an incumbent of a category such as student, mother, or friend, they make inferences regarding the rights and responsibilities, typical conduct and motives, and possibly personal characteristics of the incumbent” (Pomerantz and Mandelbaum, 2005: 152).

In the following example, the speaker initiates a proposal in lines 06–08. When there is no verbal response in line 09, the proposer produces an intimate address term, which seems to transform the original proposal into a request to some extent, thus invoking the responsibility of a brother to accept the original proposal produced by his little sister. Therefore, the prior deontic stance has been decreased when the original proposal has been altered into one that needs the recipient's permission, rather than something that can be jointly decided by both sides.

**Table:** 

(6)	**19H_XZ**	
06	Jing: →	*.h zanmen xianzai jiu zou wa. xianzai qu na*. .h We now just go PRT. Now go get. **Let's go now. Go and get it now**.
07	→	*qu Rencai Shichang nashang wa. ni shuo le*. Go Talent Market get PRT. You say PRT. **Go to Talent Market and get it. How do you think**.
08	→	*wo- xiawu huilai hai neng shuishangyijiao le*. I- afternoon back still can have a nap PRT. **Having a nap is still possible after we are back from Talent Market this afternoon**.
09		(1.2)
10	Jing: →	*gege*. Brother. **Brother**.
11		(.)
12	Quan:	*ao. keyi*. Yeah. Can. **Yeah. This can be done**.

In example (6), the wife (Jing) is calling her husband (Quan) to propose going out and retrieving an archive right now in lines 06–07. The proposal is performed with an account in line 08 which includes a self-repair transforming a possible self-attentiveness account (abandoned as the cut-off in line 08 indicates) into an other-attentiveness account using a nonperson reference design, which could invoke the speaker and the recipient as the beneficiaries of the proposal (as they could both have a rest after retrieving the archive). After a long silence of 1.2 s in line 09, the wife produces an intimate address term “*gege*.” in line 10, which indicates their relationship in terms of the rights and responsibilities between them. Thus, the husband is supposed to indulge his wife by doing what his wife asks him to do. Then immediately in the next turn (line 12), the husband acknowledges that address term and grants what the wife pursues with “*keyi.*”, which indicates his higher deontic rights. Therefore, the intimate address term used by the wife transforms the action from proposing to requesting, making the original proposal sound like something that requires the husband's permission, thus giving the husband more rights to decide. In the meanwhile, the wife's prior deontic stance has been made more tentative.

In conclusion, the proposer would make a revised other-attentiveness proposal to try to satisfy both participants. Also, by providing an account, the proposer can make the original proposal more justified, more convincing, and easier to be accepted by the recipient. Additionally, the proposer can also lower his/her prior deontic stance by pursuing with a tag question or can grant the recipient more deontic rights by requesting with an intimate address term. These subsequent actions all make the proposer's prior deontic stance more tentative, helping to pursue a preferred response or at least to mobilize an articulated one from the recipient.

### Making the Prior Deontic Stance More Decisive

It is observed that the prior deontic stance can be made more decisive by making a further arrangement, closing the local sequence, and providing a candidate unwillingness account.

#### Making a Further Arrangement

People make arrangements to complete agreed decisions, such as a request, an offer, or a proposal. Thus, the whole sequence, e.g., a whole proposal sequence, is “achieved across a series of adjacency pairs, which are nonetheless being managed as a coordinated series that overarches its component pairs” (Heritage and Sorjonen, 1994: 4). Nevertheless, when there is no such agreed decision, the proposer would still propose a further arrangement.

In example (7), the husband (Wei) is calling his wife (Jiu) to ask if she wants to go out with him to get their car washed and to buy some fish food. The wife agrees but she has to put the baby to bed first. Then, the husband asks how long it will take. The wife does not give a direct answer. Instead, she tells her husband she is still feeding the baby right now, indicating that she cannot provide a precise time as the answer to his question. However, the husband still pursues by providing a candidate time and asks for his wife's confirmation in line 20, and then comes the following talk.

**Table:** 

(7)	**18H_XCYS**	
20	Wei:	*na guji yihui shilaifenzhong ni neng bu neng qilai le*. Then probably later ten more minute you can not can get up PRT. **Then can you probably get up later in about ten minutes or not?**
21		(0.6)
22	Jiu:	*en, ao. wo chi le fan*. Uhm, oh. I finish PRT meal. **Uhm, oh. After I finish the meal**.
23		(0.3)
24	Jiu: →	*zanmen dagai:, 20 fenzhong ba*. We about:, twenty minitue PRT. **Let's meet in about 20 minutes**.
25		(0.4)
26	Jiu: →	*beimen jian. ha*. North gate meet. Ok. **Let's meet at the north gate. Ok**.
27		(0.3)
28	Wei:	*ao. 20 fenzhong ha. en. zhidao la*. Ok. 20 minute PRT. Yeah. know PRT. **Ok. In 20 minutes. Yeah. I got it**.

After the husband gets her answer in line 22, which is somewhat vague: “*wo chi le fan*.”, there is a silence of 0.3 s in line 23. Then, the wife proposes that they can meet each other in approximately 20 min in line 24. The husband does not immediately respond to the proposal, since there is a silence of 0.4 s in line 25. Nevertheless, the wife continues to make a further new proposal about the meeting location, using an imperative ending with the particle “*ha*.” in line 26 to solicit the recipient's agreement or confirmation (Cui, 2011). After another 0.3 s in line 27, the husband accepts his wife's two proposals in line 28. Despite the projection of a dispreferred response by the silence (Pomerantz and Heritage, 2013) in line 25, probably because the time that the wife proposes is doubled as we can know from line 20, the wife continues to make a further new proposal without acquiring the husband's agreement, thus making her first proposal as an accepted one or one that does not need the husband's agreement. Therefore, the deontic stance displayed in the first proposal has been made much more decisive by leaving almost no space for the husband to turn it down. In a word, making a further arrangement without the recipient's agreement to the original proposal enables the proposer to make the prior deontic stance more decisive, thus adding an imposition to the recipient than the original proposal does.

#### Closing the Local Sequence

A proposal needs to be accepted or agreed on by the recipient before it can be fully realized, and recipients “typically make action declarations (‘yeah, let's take it') and/or positive evaluations (‘yeah, that's good')” (Stevanovic, 2012b: 843) to indicate the acceptance of a proposal. In addition, when there is no further arrangement after the recipient's acceptance of a proposal, or in other words, after the second pair part to its prior action, a sequence-closing third (SCT) such as “oh”, “okay”, and assessment would be delivered to “move for, or to propose, sequence closing” (Schegloff, 2007: 118). However, if the local sequence has been closed by the speaker before the recipient's explicit acceptance or rejection, then the recipient's rights to decide have been partially deprived at that very moment. In the meanwhile, the proposer's higher decisive deontic stance is highlighted, compared with the prior deontic stance embedded in the original proposal.

In example (8), the husband (Jun) is talking with his wife (Yun).

**Table:** 

(8)	**17YYF_ZWCF**	
96	Jun:	*↑ai↓ bu dai li hai dei li. ranhou zhe ge:*, ↑Alas↓ Not want talk still must talk. Then this one:, **Alas, though you do not want to talk with me**, **you still have to. Then this**,
97		*.hh < xiawu,> en*, .hh < afternoon,> uhm, **this afternoon, uhm**,
98		(0.7)
99	Jun: →	*meishier. xiawu zai shuo ba*. Not matter. Afternoon again talk PRT. **Don't bother. Let's talk about it this afternoon**.
100		(0.6)
101	Jun: →	*°en.°* °Yeah.° **Yeah**.

Jun proposes talking with Yun in the afternoon in line 99, after abandoning something in the middle of a telling in lines 96–97, as indicated by “*ranhou*” in line 96. Yet the silence (0.6 s) occurs right after the proposal in line 100. Then without receiving any agreement or rejection from his wife, the husband closes the local sequence with “°*en*.°” in a low voice in line 101, and it sounds like the husband is acknowledging the wife's acceptance (which obviously does not occur) of his original proposal, making it an established one without the wife's agreement. Therefore, the wife's rights to decide have been deprived at that moment. In addition, in doing so, the husband makes the prior deontic stance embedded in the original proposal more decisive, leaving no space for his wife to decide at that very moment.

#### Providing a Candidate Unwillingness Account

First actions, such as requests and invitations, inherently prefer affiliative responses. When disaffiliative responses are produced, some remedial work will be performed, in that these responses will threaten or damage interpersonal relationships between participants. Also, accounts are frequently offered as a remedy for doing disaffiliative actions (Heritage, 1984a; Schegloff, 2007; Pomerantz and Heritage, 2013). Regularly, recipients tend to provide a “no fault” account (Heritage, 1984a: 272) instead of an unwillingness account. For instance, such accounts as (s)he has already got an appointment, or (s)he is under the weather, etc. are treated as no-fault ones to reject or disagree with the speakers' first actions. However, in the following example, it is the first speaker (the proposer) rather than the recipient that provides a candidate unwillingness account when the silence occurs.

In example (9), a couple is discussing dinner arrangements.

**Table:** 

(9)	**19H_HGYY**	
22	Quan: →	*.hhhh hhh. chi ge huoguo qu?* .hhhh hhh. Eat one hotpot go? **Let's eat hotpot**.
23		(3.2)
24	Quan: →	*bu [xiang chi?* Not [want eat? **You don't want to?**
25	Jing:	*[qu naer ya?* [Go where PRT? **Where do we go to eat?**
26		*.hh bu shi me:. qu naer le me*. .hh Not be PRT:. go where PRT PRT? **No. Where do we go to eat?**

In line 22, the husband (Quan) proposes eating hotpot with his wife (Jing). However, Jing does not give an immediate response, and a significantly long silence (3.2 s) occurs in line 23, which may be a harbinger of a dispreferred response. At this moment, the husband provides a candidate unwillingness account in line 24. Grammatically, the question “*bu xiang chi?*” is designed as a polar question, which projects a response of a particular lexical item like “yes” or “no,” or other equivalents or a type-conforming response (Raymond, 2000, 2003). If the wife does acknowledge the candidate unwillingness account provided by the husband, then disaffiliativeness would arise, in that the wife's unwillingness will hinder the achievement of the proposal. On the contrary, the wife would probably not agree with the candidate unwillingness account so as not to indicate disaffiliativeness. In this regard, the action conducted by the husband may impose the wife to accept the proposal, making the prior deontic stance embedded in the original proposal more decisive. Observably, the wife denies the husband's inquiry about the candidate unwillingness account in line 26, thus indicating her acceptance of the husband's imposition.

Therefore, to make the recipient accept the original proposal, the proposer would make a further arrangement or close the local sequence to ignore the silence or the absence of an immediate response from the recipient, or would provide a candidate unwillingness account to impose the recipient to accept. Also, these actions make the prior deontic stance embedded in the original proposal more decisive.

### Canceling the Prior Deontic Stance

In a proposal sequence, when there is silence or the absence of an immediate response, the proposer may withdraw his/her proposal with a counter-like action, and at the same time, cancels his/her prior deontic stance. In interaction, the SPP of an adjacency pair is not always produced after the FPP (Schegloff, 2007). Between them, there often occur insert expansions or even counters that are a special kind of alternative to the SPP. Counters “do not serve to defer the answering of the question; they replace it with a question of their own. They thus reverse the direction of the sequence and its flow; they reverse the direction of constraint” (Schegloff, 2007: 17). However, the following example suggests that the proposer rather than the recipient would do the so-called “counter” or counter-like action to address the absence of an immediate response.

In example (10), the husband (Wei) calls to tell his wife (Jiu) to meet in a while. After they have made the arrangement, the wife proposes eating dumplings at home in line 29.

**Table:** 

(10)	**18H_BMJZ**	
29	Jiu:	*zanmen zaijia zhu jiaozi chi?* We at home cook dumpling eat? **Let's cook dumpling and eat at home?**
30		(0.9)
31	Wei: →	*ao. huozhe shi jieshang nimen zai kaolv*. Oh. Or be pick up you then consider. **Oh. Or after picking you up we can then discuss**.
32		(0.2)
33	Wei: →	*sha dou [xing*. What all [ok. **Everything is ok**.
34	Jiu:	[*en. xing. na jiu xianzhe*. [Yeah. Ok. Then just be this. **Yeah. Ok. Then just call it a day**.

Receiving a silence of 0.9 s in line 30, the husband responds merely with an acknowledgment token “*ao*.” in line 31, which is not enough to indicate a full acceptance of a proposal. Then, in the same turn, the husband proposes discussing this issue when they meet. But the wife does not make an immediate response. Then, after a silence of 0.2 s in line 32, the husband withdraws his proposal with the employment of “*sha dou xing*.” in line 33 designed as an Extreme Case Formulation (Pomerantz, 1986), which literally indicates that the husband would accept whatever the wife proposes. Thus, the husband abandons his deontic rights to decide and reverses the rights to his wife with a counter-like action. In doing so, his prior deontic stance is canceled.

Thus, giving up his/her own deontic rights to decide by withdrawing the original proposal, the proposer actually transfers the rights as well as the obligations originally shared and fulfilled by both participants to the recipient.

## Results

The above examples illustrate that the relatively long silence or the absence of an immediate response is ascribed by the proposer as a harbinger of either interactional problems or potential dispreferred responses. Generally, the proposer would take subsequent actions to address them. These actions serve to pursue a preferred response or mobilize at least an articulated one. In the meanwhile, the accomplishment of these actions modulates the prior deontic stance, and the underflow of the deontic stance displays a decisive-to-tentative gradient, which can be reflected in the following figure.

**Figure 1:**
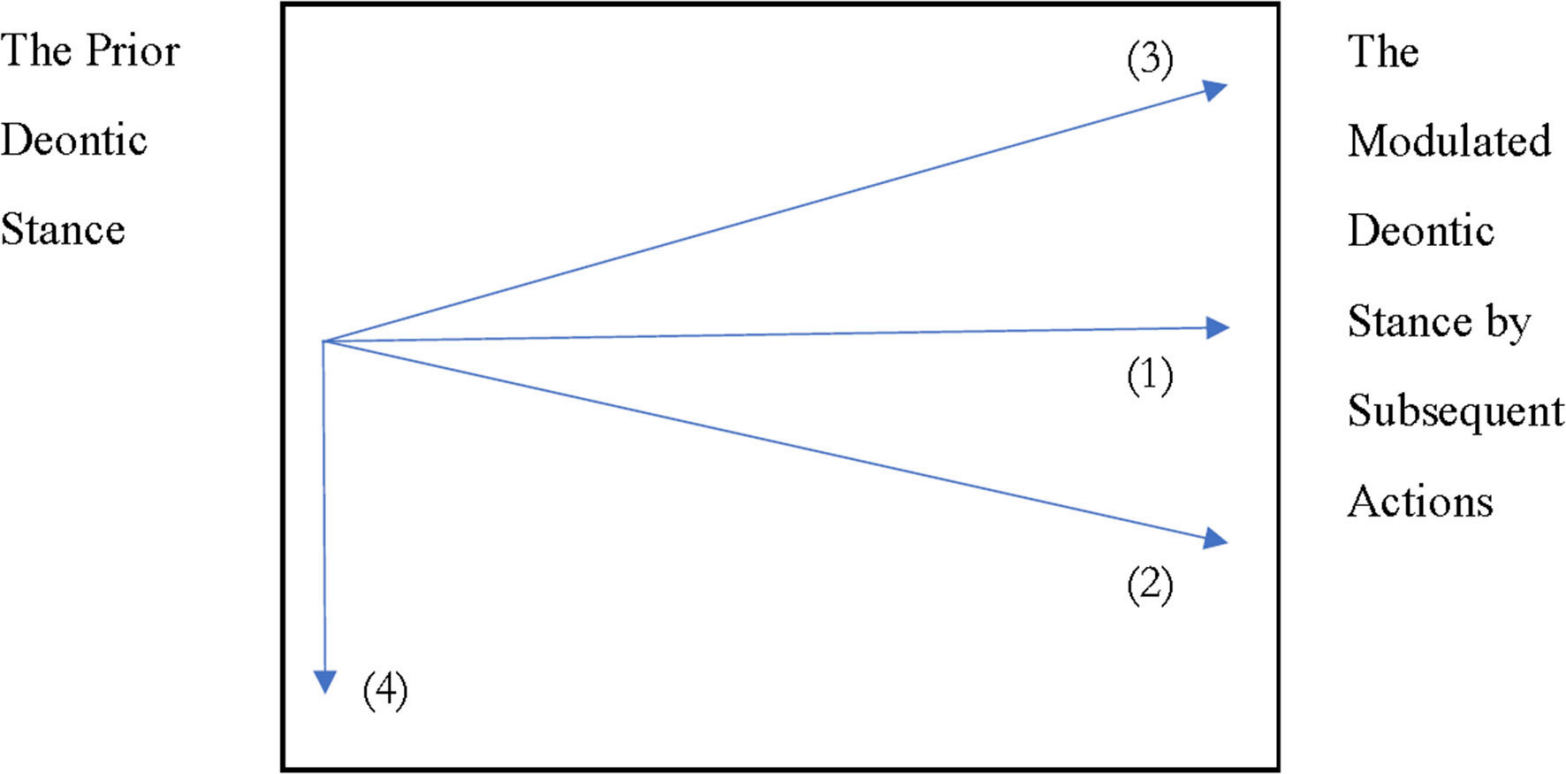
The underflow of deontic stance in proposal sequences [Lines (1)–(4) stand for the four deontic trends modulated by subsequent actions proposers do after the absence of an immediate response].

Figure 1 shows that the prior deontic stance can be maintained [line (1)], made more tentative [line (2)] or more decisive [line (3)], or even canceled [line (4)]. Moreover, it should be highlighted that what the present investigation focuses on is how the prior deontic stance is modulated by subsequent actions, but not on the deontic stance displayed by subsequent actions themselves.

Additionally, the prior deontic stance shown on the left side of Figure 1 is not invariable but dynamically constructed through various interactional resources. For example, *yàobù*-TCUs are found to be “a conversational practice which enables the proposer to tentatively make a proposal with minimal imposition on the recipient” (Yu and Hao, 2020: 18). However, for the consideration of convenience, the start point of the figure is made fixed.

## Discussion

Sequentially, the proposer takes subsequent actions to address the absence of an immediate response. Interactionally, the consequence these actions bring about is the modulation of the prior deontic stance, which has something to do with face-saving or face-threatening. Also, “face,” including positive face and negative face, has been identified and elaborated as basic human desires and characteristics of all competent adults (Goffman, 1967; Brown and Levinson, 1987). Specifically, “negative face refers to the desire to be free from imposition and to have one's autonomy and prerogatives honored and respected. Positive face refers to the desire to have a favorable self-image that is validated by others” (Clayman, 2002: 231). This study argues that maintaining the prior deontic stance damages neither proposer's nor recipient's faces; whereas, making the prior deontic stance more tentative weakens the proposer's deontic rights to decide, thus highlighting his/her damaged negative face; in contrast, making the prior deontic stance more decisive constrains the recipient's rights to make a decision so as to damage his/her negative face; and canceling the prior deontic stance actually transfers the rights as well as the obligations originally shared and fulfilled by both participants to the recipient, thus damaging the proposer's positive face as a responsible participant, as well as the recipient's negative face as a willing participant to shoulder the obligations. In our data, only in one case out of the total 34 cases, the proposer cancels the prior deontic stance, which indicates that normally the proposer would not do it. Also, this suggests that canceling may not be an appropriate action.

Besides, it is worth mentioning that the relationship between a proposer and his/her recipient is not fixed but interactionally constructed. In addition, the recipient's response plays a vital role. The modulated deontic stance, whether tentative or decisive, needs to be acknowledged by the recipient in reaching an agreement as well as building social solidarity between the two interlocutors.

## Data Availability Statement

The original contributions presented in the study are included in the article/supplementary material, further inquiries can be directed to the corresponding author/s.

## Author Contributions

All authors listed have made a substantial, direct, and intellectual contribution to the work and approved it for publication.

## Conflict of Interest

The authors declare that the research was conducted in the absence of any commercial or financial relationships that could be construed as a potential conflict of interest.

## Publisher's Note

All claims expressed in this article are solely those of the authors and do not necessarily represent those of their affiliated organizations, or those of the publisher, the editors and the reviewers. Any product that may be evaluated in this article, or claim that may be made by its manufacturer, is not guaranteed or endorsed by the publisher.

## Footnotes

^1^Levinson (1983: 298) concluded that “At each initial recognizable end of a turn-constructional unit (TCU) or transition relevance place (TRP), current speaker can select a recipient to talk next (Rule 1a); or current speaker doesn't select a recipient as next, then any (other) party may self-select, and the first one gaining the rights to the next turn (Rule 1b); or current speaker has not selected a next and no other party self-selects under Rule 1b, then current speaker may (but need not) continue (Rule 1c). Rule 2 means when rule 1c has been applied by current speaker, then at the next TRP rule 1a-c apply, and recursively at the next TRP, until speaker change is effected”.

^2^The term “silence” is more generally used, while a gap is used before a subsequent application of Rules 1b or 1c, and a lapse is used on the nonapplication of Rules 1a, 1b, and 1c).
